# Neuromuscular blocking agents in acute respiratory distress syndrome: a systematic review and meta-analysis of randomized controlled trials

**DOI:** 10.1186/cc12557

**Published:** 2013-03-11

**Authors:** Waleed Alhazzani, Mohamed Alshahrani, Roman Jaeschke, Jean Marie Forel, Laurent Papazian, Jonathan Sevransky, Maureen O Meade

**Affiliations:** 1 Department of Medicine, McMaster University Medical Centre, 1200 Main Street West, Hamilton, Ontario, L8N 3Z5, Canada; 2 Department of Critical Care and Emergency Medicine, King Fahad Hospital, Aqrabia Street, Alkhober, 31952, Kingdom of Saudi Arabia; 3 Department of Clinical Epidemiology and Biostatistics, McMaster University Medical Centre, 1200 Main Street West, Hamilton, Ontario, L8N 3Z5, Canada; 4 Service de Réanimation des Détresses Respiratoires et Infections Sévères, Assistance Publique Hôpitaux de Marseille, URMITE CNRS-UMR 6236, Aix-Marseille University, Marseille, 13015, France; 5 Division of Pulmonary and Critical Care Medicine, Johns Hopkins University, 5501 Hopkins Bayview Circle, Baltimore, MD 21224, USA

## Abstract

**Introduction:**

Randomized trials investigating neuromuscular blocking agents in adult acute respiratory distress syndrome (ARDS) have been inconclusive about effects on mortality, which is very high in this population. Uncertainty also exists about the associated risk of ICU-acquired weakness.

**Methods:**

We conducted a systematic review and meta-analysis. We searched the Cochrane (Central) database, MEDLINE, EMBASE, ACP Journal Club, and clinical trial registries for randomized trials investigating survival effects of neuromuscular blocking agents in adults with ARDS. Two independent reviewers abstracted data and assessed methodologic quality. Primary study investigators provided additional unpublished data.

**Results:**

Three trials (431 patients; 20 centers; all from the same research group in France) met inclusion criteria for this review. All trials assessed 48-hour infusions of cisatracurium besylate. Short-term infusion of cisatracurium besylate was associated with lower hospital mortality (RR, 0.72; 95% CI, 0.58 to 0.91; *P *= 0.005; *I*^2 ^= 0). This finding was robust on sensitivity analyses. Neuromuscular blockade was also associated with lower risk of barotrauma (RR, 0.43; 95% CI, 0.20 to 0.90; *P *= 0.02; *I*^2 ^= 0), but had no effect on the duration of mechanical ventilation among survivors (MD, 0.25 days; 95% CI, 5.48 to 5.99; *P *= 0.93; *I*^2 ^= 49%), or the risk of ICU-acquired weakness (RR, 1.08; 95% CI, 0.83 to 1.41; *P *= 0.57; *I*^2 ^= 0). Primary studies lacked protracted measurements of weakness.

**Conclusions:**

Short-term infusion of cisatracurium besylate reduces hospital mortality and barotrauma and does not appear to increase ICU-acquired weakness for critically ill adults with ARDS.

## Introduction

Acute respiratory distress syndrome (ARDS) is a common and life-threatening condition that complicates a variety of critical illnesses, including sepsis, pneumonia, and trauma. Characterized by intense lung inflammation, consolidation, and progressive microatelectasis, ARDS is associated clinically with severe hypoxemia, patient-ventilator dyssynchrony, and high susceptibility to barotrauma and ventilator-induced lung injury. Approximately 140,000 patients are affected by ARDS annually in the United States alone [1]. Despite advances in the relevant technology and research methods, mortality from ARDS remains as high as 26% to 58% [2,3].

Although relatively few interventions may improve survival for patients with ARDS, the interventions with most supportive research findings are ventilation strategies that aim to minimize ventilator-induced lung injury. In a landmark clinical trial, low-tidal-volume ventilation was found to improve survival for critically ill adults with acute lung injury or ARDS [4], and a systematic review of 10 related randomized trials supports this finding [5]. Whereas a lung-protective role for high levels of PEEP in adult ARDS is less clear, a patient-level meta-analysis including 2,299 participants from three trials suggests lower mortality, particularly for moderate to severe ARDS [6]. Prone ventilation may also have a lung-protective effect [7]. Similarly, neuromuscular blocking agents (NMBAs) may have an important role in the management of critically ill adults with ARDS.

Clinicians commonly rely on NMBAs in the management of ARDS to prevent patient-ventilator dyssynchrony, to minimize the work of breathing, and to improve oxygenation [8-11]. Indeed, an early, small, randomized trial demonstrated improved oxygenation with continuous cisatracurium therapy [12]. In a subsequent trial, the same group of investigators found a statistically significant reduction in inflammatory biomarkers in both the blood and bronchoalveolar fluid of patients treated with cisatracurium, along with improved oxygenation [13]. Recently, they reported a trial of 339 patients that did not show a statistically significant reduction in crude hospital mortality [14]. These potential benefits must be weighed against prevailing concerns about NMBA therapy, including progressive atelectasis due to loss of diaphragmatic tone (with resultant hypoxemia) and, most important, ICU-acquired weakness [15,16]. Those concerns previously led one guideline panel to suggest NMBAs as a consideration only in the setting of severe hypoxemia [17], and another to recommend avoiding NMBAs [18].

Given the uncertain role for NMBAs in the management of adults with ARDS, we conducted a systematic review and a meta-analysis, including previously unpublished data, to clarify the effects of NMBA on mortality and other clinically important outcomes.

## Materials and methods

By following a prespecified research protocol, this review included parallel-group randomized trials investigating the administration of any NMBA to mechanically ventilated adults with ARDS, as defined by American-European Consensus Conference (AECC) [19], regardless of the underlying etiology, and dating back to 1966. Outcomes of interest included measures of mortality at 28 days, ICU discharge, and hospital discharge (primary outcome); duration of mechanical ventilation (in all patients and in survivors), ventilator-free days (VFDs), ICU and hospital stay; changes in oxygenation (measured by using the PO_2_/FiO_2 _ratio); ICU-acquired weakness; and barotrauma (including pneumothorax, pneumomediastinum, pneumatocele, and subcutaneous emphysema).

### Search strategy

Computerized literature searches included MEDLINE (1966 to October 2012), EMBASE (1980 to October 2012), ACP Journal Club (1991 to October 2012), the Cochrane (Central) database, and clinical trial registries (clinicaltrials.gov, ISRCTN Register, and WHO ICTRP) (see Additional file 1). Relevant conference proceedings were searched electronically through a specific service provided through McMaster University [20].

### Study selection and data extraction

Two reviewers (WA and MA) independently screened titles and abstracts in duplicate, without language restriction. The same duplicate, independent review process was followed by reviewing the full text of all potentially eligible articles; by reviewing citation lists of these articles for additional studies; and abstracting data (related to outcomes, or to risk of bias) onto customized, pretested forms. To resolve disagreements, we contacted study authors. For the purposes of this review, study authors provided unpublished data related to hospital mortality (truncated at 90 days), duration of mechanical ventilation, VFDs, and gas exchange.

### Assessment of risk of bias of included studies

For each trial, reviewers used the Cochrane Risk of Bias tool to judge the adequacy of randomization, concealment, blinding, and outcome-data completeness, and to check for selective outcome assessments and other possible sources of bias [21]. Reviewers judged the risk of bias in each of these domains as high risk, low risk, or unclear. The overall risk of bias for an individual trial was categorized as low when the risk of bias was low in all domains; unclear when the risk of bias was unclear in at least one domain, with no high-risk domains; or high when the risk of bias was high in at least one domain.

### Statistical analysis

We pooled data by using RevMan 5.1 and random-effects models, applying inverse variance weighting and the methods of DerSimonian and Laird [22]. We generated summary estimates of relative risk (RR) for dichotomous outcomes and mean differences (MDs) for continuous outcomes, each with associated 95% confidence intervals (CIs). To investigate the effect of treatment on oxygenation, we analyzed early changes in PaO_2_/FiO_2 _at 24, 48, and 72 hours after randomization. To assess for effects on duration of ventilation, we planned to compare VFDs at day 28. Because of the controversy surround VFDs as an outcome, we conducted *post hoc *comparisons of duration of ventilation in survivors, and all patients. We assessed for heterogeneity between studies by using the Mantel-Haenszel χ^2 ^statistic (*P *< 0.01 indicating substantial heterogeneity) and the *I^2 ^*statistic (> 50% indicating substantial heterogeneity). We had too few studies to assess for publication bias by using a funnel plot or conventional statistical methods [23].

The review protocol stipulated a number of exploratory analyses to assess potential reasons for differing results (if any) across studies. We hypothesized that two factors might generate estimates of greater benefit: high or unclear risk of bias (versus low), and more-severe hypoxemia at baseline (PO_2/_/FiO_2 _< 100, versus 100 to 200). To inform current guidelines, we also sought to estimate the effect of NMBA specifically among sepsis patients. With RevMan 5.1 software, subgroup analyses were conducted by pooling RRs for subgroups in each trial. Last, we planned to test the robustness of our primary (mortality) results in sensitivity analyses by using fixed-effects models and by using two alternative statistical metrics: odds ratios and risk differences. To estimate the number of adults with ARDS that must receive NMBA therapy to save one additional life (number need to treat), we assumed a mortality rate of 40% in the absence of NMBA therapy, in accordance with mortality rates in current trials [14].

After judging the risk of bias for each study, and pooling results across studies, we judged the quality of the totality of the evidence addressing the role for NMBAs in the management of ARDS. We used the Grading of Recommendations Assessment, Development and Evaluation (GRADE) approach, which considers (a) risk of bias in individual trials, (b) consistency of results across trials), (c) potential for publication bias, (d) precision of pooled estimates, and (e) suitability of the individual study populations, interventions, and outcome assessments in directly addressing the question of this review [24].

## Results

**Figure 1:**
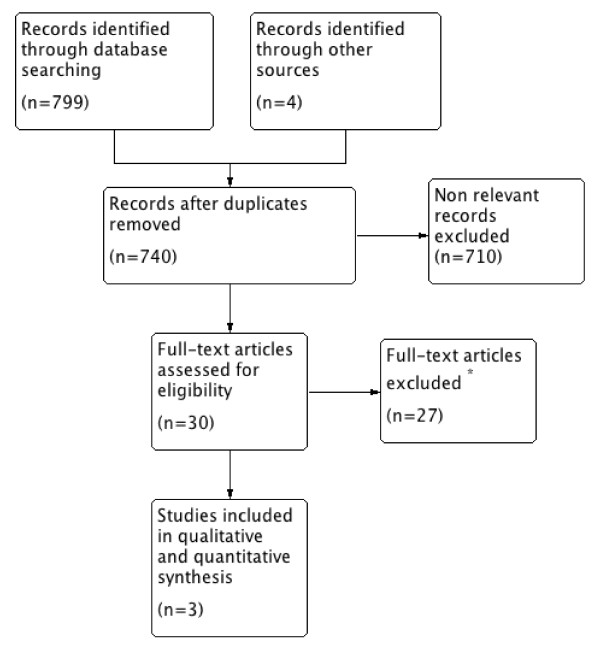
**Summary of evidence search and selection**. Flow diagram showing steps of study selection.

After screening 740 titles and abstracts, we found three trials eligible for this review (Figure 1). All three trials originated from the same group of investigators, including a total of 431 patients, and one trial was conducted in 20 centers in France. The trials were specifically designed to investigate the effects of cisatracurium besylate, a benzylisoquinoline compound, on gas exchange [12], inflammatory markers [13], and clinically important outcomes [14], respectively. In each trial, cisatracurium besylate was infused for 48 hours, by using weight-based dosing in two trials [12,13], and a fixed high dose (15 mg bolus, followed by a continuous infusion of 37.5 mg per hour) in the other (Table 1) [14]. All three trials reported 28-day mortality, barotraumas, and ICU-acquired weakness; and these data were supplemented by additional, previously unpublished data from the study investigators.

**Table 1:** Characteristics of included trials

Study	Number (sites)	Target patients	Experimental intervention	Control intervention	Lung protection
Gainnier 2004 [12]	56	ARDS	48-hour infusion cisatracurium	48-hour infusion placebo (bedside nurse not blinded)	ARMA protocol; no weaning protocol
	(4)	PaO_2_/FiO_2 _< 150	(weight-based, and adapted to peripheral nerve stimulation)		
		Eligible < 36 hours			
		Exclude prior NMBA			

Forel 2006 [13]	36	ARDS	48-hour infusion cisatracurium	48-hour infusion placebo (bedside nurse not blinded)	ARMA protocol; no weaning protocol
	(3)	Intubated < 48 hours	(weight-based and adapted to peripheral nerve stimulation)		
		PaO_2_/FiO_2 _< 200			
		Exclude recent steroids or NMBA			

Papazian 2010 [14]	340	ARDS	48-hour infusion cisatracurium	48-hour infusion placebo	ARMA protocol; weaning protocol
	(20)	PaO_2_/FiO_2 _< 150	(high-dose, with no peripheral nerve stimulation)		
		Eligible < 48 hours			
		Exclude prior NMBA			

ARDS, Acute respiratory distress syndrome; NMBA, neuromuscular blocking agent; ARMA, the Acute Respiratory Distress Syndrome Network. Ventilation with lower tidal volumes as compared with traditional tidal volumes for acute lung injury and the acute respiratory distress syndrome [4].

In terms of the quality of individual trials, one trial had low risk of bias [14]; the other two were judged to be at high risk of bias because of limitations in blinding (Table 2). In each trial, physicians and nurses ascertained that study patients were deeply sedated (with no response to glabellar tap) before initiating the study infusion. In two trials, the study drug-infusion bag (containing either cisatracurium besylate or normal saline) was concealed by a sheet, and the only caregivers explicitly aware of treatment allocation were bedside nurses caring for study participants during the 48 hours of study infusion. Nurses were responsible for the assessment of neuromuscular blockade (at 8-hour intervals during the study infusion and the following 24 hours) and for the protocolized delivery of sedation and cisatracurium besylate. The most recent trial included a more-robust placebo. Study infusion bags contained identical solutions of cisatracurium besylate or normal saline, prepared outside of the hospital; peripheral nerve stimulators were not used to assess depth of paralysis; nurses assessed plateau airway pressure to determine whether additional study drug was required.

**Table 2:** Methodologic quality of trials

Study	Sequence generation	Allocation concealment	Blinding	Withdrawal; loss to follow-up	Selective outcome reporting	Free of other bias	Overall risk of bias
Gainnier 2004 [12]	Low risk of bias	Low risk of bias	High risk of bias	Low risk of bias	Low risk of bias	Low risk. of bias	High
	Computer-generated random number sequences	Centralized	Nurses aware of assignment; infusion covered by sheet	None	None	None	

Forel 2006 [13]	Low risk of bias	Low risk of bias	High risk of bias	Low risk of bias	Low risk of bias	Low risk. of bias	High
	Computer-generated random number sequences	Centralized	Nurses aware of assignment; infusion covered by sheet	None	None	None	

Papazian 2010 [14]	Low risk of bias	Low risk of bias	Low risk of bias	Low risk of bias	Low risk of bias	Low risk. of bias	Low
	Computer-generated random number sequences	Centralized, using undisclosed block sizes	Blinding of patients, clinicians, evaluators, investigators, analysts	None	None	None	

**Figure 2:**
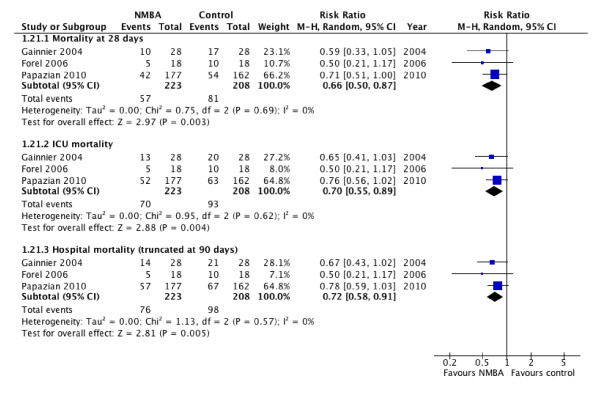
**Mortality**. Forest plot comparing neuromuscular blockers and placebo for the following outcomes: 28 days, ICU, and hospital (truncated at 90 days), results are shown by using random-effects model with relative risk and 95% confidence interval.

Cisatracurium besylate was associated with lower risk of death at 28 days (RR, 0.66; 95% CI, 0.50 to 0.87; *P *= 0.003; *I*^2 ^= 0; Figure 2), at ICU discharge (RR, 0.70; 95% CI, 0.55 to 0.89; *P *= 0.004; *I^2 ^*, 0; Figure 2), and at hospital discharge (RR, 0.72; 95% CI, 0.58 to 0.91; *P *= 0.005; *I*^2 ^= 0; Figure 2). Assuming a mortality rate of 40% in the absence of cisatracurium therapy at each of these time points, these pooled estimates suggest a number needed to treat of seven patients (95% CI, 5 to 19) to save one additional life at 28 days; eight patients (95% CI, 4 to 31) to save one additional life at ICU discharge; and nine patients (95% CI, 6 to 27) for hospital mortality (Table 3).

**Table 3:** Summary of pooled results

End point (outcome)	Number of trials (number of patients)	Number of events in each group (%)	**Results**^a^	Absolute effect per 1,000 treated patients^b^	Quality of evidence
Hospital mortality	3 (431)	Intervention: 76/223 (34%)	RR, 0.72 (CI, 0.58 to 0.91); *P *= 0.005; *I*^2 ^= 0	132 fewer per 1,000 (from 42 fewer to 198 fewer)	Moderate^c^
		Control: 98/208 (47%)			

ICU mortality	3 (431)	Intervention: 70/223 (31.4%)	RR, 0.70 (CI, 0.55 to 0.89); *P *= 0.004; *I*^2 ^= 0	134 fewer per 1,000 (from 49 fewer to 201 fewer)	Moderate^c^
		Control: 93/208 (44.7%)			

Mortality at 28 days	3 (431)	Intervention: 57/223 (25.6%)	RR, 0.66 (CI, 0.50 to 0.87); *P *= 0.003; *I*^2 ^= 0	132 fewer per 1,000 (from 51 fewer to 195 fewer)	Moderate^c^
		Control: 81/208 (39%)			

Days free of mechanical ventilation at 28 days	3 (431)	n/a	MD, 1.91 (CI, 0.28 to 3.55); *P *= 0.02; *I*^2 ^= 0	n/a	Moderate^c^

Duration of mechanical ventilation	3 (431)	n/a	MD, 1.21 (CI, 4.23 to 1.81); *P *= 0.43; *I*^2 ^= 0	n/a	Moderate^c^

Barotrauma	3 (431)	Intervention: 9/223 (4%)	RR, 0.43 (CI, 0.20 to 0.90); *P *= 0.02; *I*^2 ^= 0	55 fewer per 1,000 (from 10 fewer to 77 fewer)	Moderate^c^
		Control: 20/208 (9.6%)			

ICU Acquired weakness	3 (431)	Intervention: 73/223 (32.7%)	RR, 1.08 (CI, 0.83 to 1.41); *P *= 0.57; *I*^2 ^= 0	24 more per 1,000 (from 51 fewer to 122 more)	Very weak^cde^
		Control: 62/208 (30%)			

ICU Length of stay	1 (339)	n/a	MD, 1.80 (CI, 5.93 to 2.33); *P *= 0.39	n/a	n/a

CI, confidence interval; ICU, intensive care unit; MD, mean difference; n/a, not applicable; RR, risk ratio. ^a^Pooled relative risk among RCTs. **^b^**The corresponding risk (and its 95% confidence interval) is based on the assumed risk in the comparison group and the relative effect of the intervention (and its 95% CI). ^c^Rated down for incomplete blinding. ^d^Rated down for ascertainment bias (limited assessment of weakness in two trials). ^e^Rated down for imprecision. GRADE, Working Group grades of evidence; High quality, further research is very unlikely to change our confidence in the estimate of effect; Moderate quality, further research is likely to have an important impact on our confidence in the estimate of effect and may change the estimate; low quality, Further research is very likely to have an important impact on our confidence in the estimate of effect and is likely to change the estimate; very low quality, we are very uncertain about the estimate.

**Figure 3:**
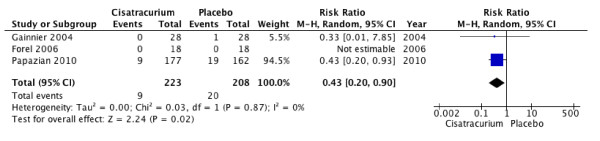
**Barotrauma**. Forest plot comparing neuromuscular blockers and placebo for barotrauma outcome; results are shown by using random-effects model with relative risk and 95% confidence interval.

**Figure 4:**
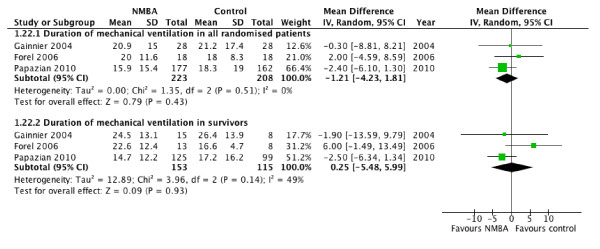
**Duration of mechanical ventilation**. Forest plot comparing neuromuscular blockers and placebo for the duration of mechanical ventilation in all patients and in survivors; results are shown by using random-effects model with relative risk and 95% confidence interval.

NMBA therapy was also associated with lower risk of barotrauma (RR, 0.43; 95% CI, 0.20 to 0.90; *P *= 0.02; *I*^2 ^= 0; Figure 3), and increased VFDs over a period of 28 days (MD, 1.91 days; 95% CI, 0.28 to 3.55; *P *= 0.02; *I*^2 ^= 0), but no appreciable difference in the duration of mechanical ventilation for all patients (MD, 1.21; 95% CI, 4.23 to 1.81; *P *= 0.43; *I*^2 ^= 0; Figure 4) or specifically among survivors (MD, 0.25 days; 95% CI, 5.48 to 5.99; *P *= 0.93; *I*^2 ^= 49%; Figure 4). Only one trial reported ICU length of stay, which did not differ between groups (mean difference, 1.80 days; 95% CI, 5.93 to 2.33; *P *= 0.39), even when the analysis was limited to survivors (mean difference, 2.90 days; 95% CI, 7.86 to 2.06; *P *= 0.25).

**Figure 5:**
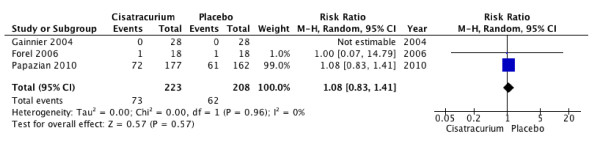
**ICU-acquired weakness**. Forest plot comparing neuromuscular blockers and placebo for ICU-acquired weakness outcome; results are shown by using random-effects model with mean difference and 95% confidence interval.

All three trials reported ICU-acquired weakness. One trial used a validated measure (Medical Research Council (MRC) score) to screen systematically for ICU-acquired weakness [14]. The other two trials used a clinical assessment of "quadriparesis" as a definition of ICU-acquired weakness. The use of NMBA was not associated with increased risk of ICU-acquired weakness (RR, 1.08; 95% CI, 0.83 to 1.41; *P *= 0.57; *I*^2 ^= 0; Figure 5). In total, 190 patients included in this review received corticosteroid therapy during the study period of cisatracurium infusion.

**Figure 6:**
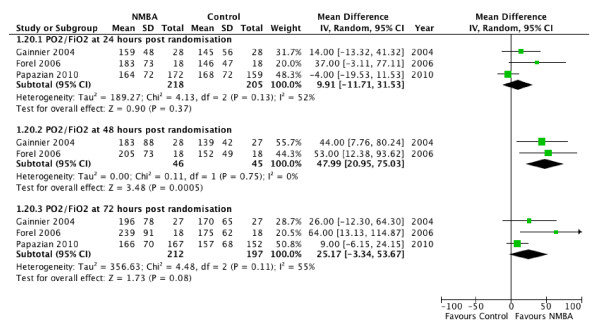
**Oxygenation at 24 to 72 hours**. Forest plot comparing neuromuscular blockers and placebo for oxygenation outcome (measured by using PaO_2_/FiO_2 _at 24 to 72 hours after randomization); results are shown by using random-effects model with mean difference and 95% confidence interval.

Oxygenation was assessed by using PaO_2_/FiO_2 _at various time points after randomization. The pooled analysis suggested better PaO_2_/FiO^2 ^in the NMBA group at 24, 48, and 72 hours after randomization, but only the result at 48 hours was statistically significant (Figure 6).

Results of this review were consistent across the three trials; however, we proceeded with planned analyses to test their robustness. With respect to the effects of NMBA on hospital mortality, we found no interactions with study risk of bias, etiology of ARDS as sepsis versus other, or with severity of hypoxemia (Table 4). Sensitivity analyses by using fixed-effects models, pooled odds ratios, and pooled absolute risk difference generated similar results, with statistically significant reductions in hospital mortality (data not shown). Although the trial by Papazian *et al. *[14] was the largest and contributed the greatest weight to the analysis of hospital mortality, the result remained statistically significant in a *post hoc *sensitivity analysis excluding this trial (RR, 0.63; 95% CI, 0.43 to 0.92; *P *= 0.02; *I*^2 ^= 0).

**Table 4:** Subgroup analyses for hospital mortality outcome

Subgroup	Number of patients (*n*)	Relative risk (95% CI)	*P *value (interaction between groups)
Methodologic quality of trials			
Low risk of bias	339	0.78 (0.59 to 1.03)	0.38
High/unclear risk of bias	92	0.63 (0.43 to 0.92)	

Cause of ARDS			
Sepsis	311	0.72 (0.54 to 0.94)	0.83
Other causes	120	0.76 (0.47 to 1.24)	

PaO_2_/FiO_2_			
≥ 100 to 200	256	0.77 (0.57 to 1.03)	0.87
< 100	175	0.74 (0.51 to 1.06)	

ARDS, acute respiratory distress syndrome; PaO_2_/FiO_2_, ratio of partial arterial pressure of oxygen to fraction of inspired oxygen.

Table 3 summarizes the quality of the totality of evidence in this review. Overall, we judged the quality of evidence related to mortality as moderate in light of the limitations in blinding, and the possibility of publication bias. We judged the quality of evidence related to ICU-acquired weakness as weak.

## Discussion

In this meta-analysis, we found that the treatment of critically ill adults with a 48-hour continuous infusion of cisatracurium besylate consistently reduced the risk of death at 28 days, ICU discharge, and hospital discharge, reduced the risk of barotrauma, and did not affect the duration of mechanical ventilation or the risk of ICU-acquired weakness.

In terms of the mortality reduction associated with NMBA therapy, our findings are large and robust. We determined that for every nine adults with ARDS receiving cisatracurium therapy, one additional life is saved during the first 90 days in hospital. This magnitude of effect is larger than that achieved with low-tidal-volume ventilation [25]. Moreover, sensitivity analyses using odds ratios or absolute risk difference produced similar statistically significant findings. Duration of mechanical ventilation was not significantly different between groups, including groups of patients who survived (Figure 4). However, VFDs were increased in the cisatracurium group, as a result of competing risks of death and duration of ventilation, both of which are integrated into this outcome.

The present systematic review builds on the similar findings of a recent review by Neto *et al. *[26]. We analyzed important new and previously unpublished data about hospital mortality, an outcome that carries more weight in clinical decision making and clinical-practice guidelines. Moreover, we present additional subgroup analyses addressing severity of hypoxemia and etiology of ARDS, as well as more-complete analyses related to the duration of ventilation.

Clinical observations and systematic research both support the notion that NMBA therapy improves oxygenation among critically ill patients with ARDS [12,27], although the mechanism leading to this effect is not entirely clear. In terms of lung mechanics, better synchrony may lead to more-uniform lung recruitment, improved compliance, better gas exchange, and better systemic oxygenation. With respect to lung inflammation, it is plausible that improved control of inspiratory volumes and pressures reduces volutrauma, while better control of expiratory volumes and pressures reduces atelectrauma; the result being less pulmonary and systemic inflammation [4]. The latter hypothesis is supported by one of the three trials included in this review, which demonstrated a significant reduction in pulmonary levels of IL-6, IL-8, and IL-1B in cisatracurium-treated patients, along with improved oxygenation [13]. Whatever the mechanism, this review found a corresponding improvement in early mortality.

A prominent criticism of this literature has been the relative lack of caregiver blinding, even in the largest and most recent trial, which was placebo controlled. In this trial, bedside clinicians confirmed the adequacy of deep sedation (defined as no response on glabellar tap) before initiating a high-dose infusion of either cisatracurium besylate or an identical-appearing placebo. Thereafter, clinicians did not monitor the depth of paralysis with peripheral nerve stimulation; rather, they monitored airway pressures and, when plateau pressures exceeded 32 cm H_2_O (for more than 10 minutes, and despite increased sedation) in either group, an open-label intravenous bolus of cisatracurium was administered. We believe that the majority of patients with severe ARDS who have no response to glabellar tap are unlikely to initiate spontaneous breaths, and, for that majority of patients, caregivers remained blinded. In the application of this protocol outside a research setting, monitoring the depth of paralysis with peripheral nerve stimulation would serve to prevent unnecessarily high dosing of cisatracurium besylate and, accordingly, possibly reduce any adverse effects.

The possibility of a link between neuromuscular blockade and risk of ICU-acquired weakness poses a strong deterrent to NMBA therapy in current management of adult ARDS [15,16,28,29]. The strongest clinical research supporting this association includes four retrospective studies (*n *= 481) in the management of severe asthma [15]. These studies were confounded by concurrent, high-dose glucocorticosteroid therapy. In contrast, slightly less than one half of the patients in this review received corticosteroid therapy during the study infusion of cisatracurium besylate, at lower doses than those administered for acute asthma. Moreover, the observational studies in asthma generally lacked systematic screening for ICU-acquired weakness. Common conclusions from the asthma literature are that (a) prolonged quadriparesis may be related to the dose and duration of neuromuscular blockade, (b) particularly in the settings of coexistent renal or hepatic dysfunction, and (c) a class effect may exist (although the data are inconsistent) with amino steroid NMBAs (for example, pancuronium, vecuronium) posing a higher risk of ICU-acquired weakness than benzylisoquinolones (for example, atracurium, cisatracurium).

In contrast to the prior literature in asthma, we reviewed randomized trials of patients with severe ARDS. Our review found no apparent increase in ICU-acquired weakness with cisatracurium therapy. The definition of this outcome in two of the three trials was based simply on clinically detectable quadriparesis [12,13], which may lack both sensitivity and specificity; however, the most recent and largest trial used the validated Medical Research Council score [28], and found identical risk of ICU-acquired weakness whether or not patients received NMBA therapy. Future studies could use the same measure over a more protracted period of time, and supplement these assessments with electrophysiologic testing.

The strengths of this review include adherence to a predetermined review protocol, a comprehensive literature search, duplicate independent judgments about study eligibility and risk of bias, and collaboration with authors of the primary studies after the review protocol had been established.

Four noteworthy limitations exist. First, it is conceivable that incomplete blinding in two trials might have led to inflated estimates of benefit. Second, we were unable to assess for publication bias. Third, it is uncertain whether the results of this review are applicable to cisatracurium besylate only, to all benzylisoquinolone agents, or to all neuromuscular blocking agents and, in addition, we were unable to investigate optimal dosing strategies. Finally, these findings may not be applicable to centers in which the care of adults with ARDS differs significantly from the care provided in France, where the 20 centers involved in these studies were located.

Current clinical management of critically ill adults with ARDS commands further direction on the role for NMBA therapy, which has become an important part of the armamentarium for severe hypoxemia [6]. In the three recent clinical trials of high PEEP for the management of adult acute lung injury and ARDS, in which NMBA therapy was not protocolized, more than one half of 2,299 patients received neuromuscular blockade at some time during their study, for a median of 2.5 days [6]. Interestingly, during a decade of randomized trials comparing low-tidal-volume ventilation with traditional tidal volumes, significantly more patients managed with low-tidal-volume strategies received NMBA therapy (RR, 1.37; 95% CI, 1.04 to 1.82; *P *= 0.030) [5], suggesting that NMBA may already play a very important role in lung-protective ventilation.

## Conclusions

In summary, results of this review suggest that a 48-hour continuous infusion of cisatracurium besylate for patients with severe ARDS reduces 28-day, ICU, and hospital mortality, as well as barotrauma, without increasing the risk of ICU-acquired weakness. Although these findings were derived from a single group of investigators at multiple centers across France, further international multicenter trials maybe warranted to confirm the generalizability of these findings. Meanwhile, many clinicians have adopted NMBA therapy into their routine management of adult ARDS. Given that mortality is the critical outcome of interest in this setting, our findings provide new support for a short-term infusion of cisatracurium besylate for critically ill adults with severe ARDS.

## Key messages

• Few published studies suggested that the use of neuromuscular blocking agents improves lung mechanics, patient-ventilator asynchrony, and reduces inflammatory mediators in patients with ARDS.

• A recently published randomized controlled trial suggested that the use of cisatracurium in patients with ARDS may reduce mortality.

• A systematic review and meta-analysis of RCTs examining the effect of using NMBA (cisatracurium) resulted in a significant reduction in risk of death at 28 days and at ICU and hospital discharge when compared with placebo or no intervention. The risk of barotrauma was reduced with no increase in the risk of ICU-acquired weakness.

## Abbreviations

ARDS: acute respiratory distress syndrome; CI: confidence interval; FiO_2_: fraction of inspired oxygen; GRADE: grading of recommendations assessment, development, and evaluation; ICU: intensive care unit; MD: mean difference; MRC score: medical research council score; NMBA: neuromuscular blocking agent; PEEP: positive end-expiratory pressure; PO_2_: partial pressure of oxygen in the blood; RR: relative risk or risk ratio.

## Competing interests

Laurent Papazian received grants for ACURASYS trial from GlaxoSmithKline and honoraria for advice or public speaking for Faron (June 2011). Other authors declare that they have no competing interests.

## Authors' contributions

WA and MA conceived the idea and designed the study. WA and MA performed data abstraction. LP and J-M F provided data from original studies. WA conducted the analysis. WA, RJ, and MM drafted the article. All of the authors critically revised the manuscript and agreed on the submitted version.

## Supplementary Material

Additional file 1 **Search strategy and references: Contains electronic database search strategy (search terms) and reference list of all excluded full-text articles that were assessed for eligibility**. Click here for file
