# Supplementary material for: Neuromuscular blocking agents in acute respiratory distress syndrome: a systematic review and meta-analysis of randomized controlled trials
Source: Crit Care. 2013 Mar 11;17(2):R43. doi: 10.1186/cc12557 (PMC3672502; doi:10.1186/cc12557)
Supplement: Additional file 1 — Search strategy and references: Contains electronic database search strategy (search terms) and reference list of all excluded full-text articles that were assessed for eligibility. [file cc12557-S1.DOCX]

**Appendix**

**Search Strategies**

1     exp lung diseases/

2     Respiratory Distress*.mp.

3     adult Respiratory Distress Syndrome.mp.

4     ARDS.mp.

5     idiopathic respiratory distress [syndrome.mp](http://syndrome.mp/).

6     transfusion related acute lung [injury.mp](http://injury.mp/).

7     shock [lung.mp](http://lung.mp/).

8     human [ards.mp](http://ards.mp/).

9     noncardiogenic pulmonary [edema.mp](http://edema.mp/).

10     increased-permeability pulmonary [edema.mp](http://edema.mp/).

11     stiff [lung.mp](http://lung.mp/).

12     acute respiratory [distress.mp](http://distress.mp/).

13     (acute lung injury or ALI).mp.

14     [pneumonia.mp](http://pneumonia.mp/).

15     hyaline membrane [disease.mp](http://disease.mp/).

16     exp respiratory insufficiency/

17     (respiratory adj1 (failure or insufficiency)).mp.

18     or/1-17

19     exp Neuromuscular Blocking Agents/

20     Neuromuscular Blocking Agent*.mp.

21     Neuromuscular Blocker*.mp.

22     curare like [activity.mp](http://activity.mp/).

23     ((curariform or curarizing) adj1 agent*).mp. [mp=protocol supplementary concept, rare disease supplementary concept, title, original title, abstract, name of substance word, subject heading word, unique identifier]

24     Neuromuscular Blocking drug*.mp.

25     (Rapacuronium or Raplon or Mivacurium or Mivacron or Atracurium or Tracrium or Doxacurium or Nuromax or Cisatracurium or Nimbex or Vecuronium or Norcuron or Rocuronium or Zemuron or Pancuronium or Pavulon or Tubocurarine or Jexin or gallamine or Flaxedil or Pipecuronium or alcuronium orcurare or toxiferine).mp.

26     or/19-25 (

27     randomized controlled [trial.pt](http://trial.pt/).

28     Randomi?ed Controlled trial*.mp.

29     Randomi?ed clinical trial*.mp.

30     Random Allocation/

31     Random [allocation.mp](http://allocation.mp/).

32     random*.tw.

33     clinical trial/

34     controlled clinical trial/

35     single-blind method/

36     double-blind method/

37     ((singl* or doubl* or trip* or trebl*) adj25 (blind* or mask*)).mp.

38     Placebos/

39     placebo*.tw.

40     drug therapy.fs.

41     trial.ab.

42     groups.ab.

43     or/27-42

44     humans/

45     animals/

46     45 not (44 and 45)

47     43 not 46

48     18 and 26 and 47

**Reference list of excluded articles**

**Observational studies (6)**

1. Cawley, M., Czosnowski, Q., & Palkovic, L. (2010). Comparison of sedatives, analgesics and neuromuscular blocker requirements during pressure control and airway pressure release ventilation. *Critical Care Medicine.Conference: 40th Critical Care Congress of the Society of Critical Care Medicine San Diego, CA United States.Conference Start: 20110115 Conference End: 20110119.Conference Publication: (Var.Pagings), 38*, A251.
2. Conti, G., Vilardi, V., Rocco, M., DeBlasi, R. A., Lappa, A., Bufi, M., et al. (1995). Paralysis has no effect on chest wall and respiratory system mechanics of mechanically ventilated, sedated patients. *Intensive Care Medicine, 21*(10), 808-812.
3. Kallet, R. H., Eisner, M., & Luce, J. M. (2001). Sedation and neuromuscular blocking agent (NMBA) requirements during initiation of low vo ventilation in patients with acute lung injury (ALI). *Respiratory Care, 46*(10), 1122.
4. Lee Hough, C. (2006). Neuromuscular sequelae in survivors of acute lung injury. *Clinics in Chest Medicine, 27*(Acute Respiratory Distress Syndrome.), 691-703.
5. Lefrant, J.-Y. ; Cuvillon, P. ; Pandolfi, J.-L. Continuous infusion of atracurium in critically ill patients with acute respiratory distress syndrome (ARDS). European Society of Anaesthesiologists 1997; british journal of anaesthesia 1997, 78 (SUPP 1): A.363.
6. Calon, B. ; Launoy, A. ; Pottecher, T. Long-term use of rocuronium in ICU patients with or without multi organ failure. European Society of Anaesthesiologists 1999; british journal of anaesthesia 1999, 82 (SUPP 1): A.547.

**Review Articles (8)**

1. Behbehani, N. A., Al-Mane, F., D'yachkova, Y., Pare, P., & FitzGerald, J. M. (1999). Myopathy following mechanical ventilation for acute severe asthma: The role of muscle relaxants and corticosteroids. *Chest, 115*(6), 1627-1631.
2. De Jonghe, B., Lacherade, J. -., Durand, M. -., & Sharshar, T. (2007). Critical illness neuromuscular syndromes. *Critical Care Clinics, 23*(Early Mobility of the ICU Patient.), 55-69.
3. Elsasser, S., Schachinger, H., & Strobel, W. (1999). Adjunctive drug treatment in severe hypoxic respiratory failure. *Drugs, 58*(3), 429-446. Emery, E. R. (1971). [Use of pancuroniumbromide in the intensive care unit]. [Der Gebrauch von Pancuroniumbromid in der Intensivstation.] *Anaesthesist, 20*(6), 237-238.
4. Forel, J. M., Roch, A., & Papazian, L. (2009). Paralytics in critical care: Not always the bad guy. *Current Opinion in Critical Care, 15*(1), 59-66.
5. Frank, A. J., & Thompson, B. T. (2010). Pharmacological treatments for acute respiratory distress syndrome. *Current Opinion in Critical Care, 16*(1), 62-68.
6. Freebairn, R., & McHugh, G. (2010). Neuromuscular blockade in the optimal management of mechanical ventilation of patients with respiratory distress. *Current Respiratory Medicine Reviews, 6*(4), 223-228.
7. Horner, D., & Cairns, C. (2011). Early neuromuscular blockade in severe ARDS. *Journal of the Intensive Care Society, 12*(2), 153-154.
8. Lagneau, F. (2008). Indications and uses of neuromuscular blocking agents in the ICU. [Indications et utilisation des curares en reanimation.] *Annales Francaises d'Anesthesie Et De Reanimation, 27*(7-8) (pp 567-573), ate of Pubaton: Juy 2008/August 2008.

**Systematic review or meta-analysis (1)**

1. Cools, F., & Offringa, M. (2009). Neuromuscular paralysis for newborn infants receiving mechanical ventilation. *Cochrane Database of Systematic Reviews, 4) , 2009*, ate of Pubaton: 2009.

**Duplicates (1)**

1. Papazian, L., Forel, J. -., Gacouin, A., Perrin, G., Jaber, S., Arnal, J. -., et al. (2009). Systematic two-day muscle relaxants course in the early phase of severe acute respiratory distress syndrome. A multicenter randomized controlled trial. *Intensive Care Medicine.Conference: 22nd Annual Congress of the European Society of Intensive Care Medicine, ESICM Vienna Austria.Conference Start: 20091011 Conference End: 20091014.Conference Publication: (Var.Pagings), 35*, S6.

**Editorial or letter (6)**

1. Devlin, J. W., Garpestad, E., & Hill, N. S. (2010). Neuromuscular blockers and ARDS. *New England Journal of Medicine, 363*(26), 2562-4.
2. Gusmao, D. (2010). Neuromuscular blockers and ARDS. *New England Journal of Medicine, 363*(26), 2562-2563.
3. Sevransky J. ACP Journal Club. 48 hours of cisatracurium reduced 90-day mortality in patients with early, severe ARDS. *Ann Intern Med. 2011 Jan 18;154(2):JC1-3.*
4. Pathak, V., & Islam, T. (2011). Neuromuscular blockers improves outcome in severe and early adult respiratory distress syndrome. *Clinical Pulmonary Medicine, 18*(2), 95.
5. Puthucheary, Z., Hart, N., & Montgomery, H. (2010). Neuromuscular blockers and ARDS. *New England Journal of Medicine, 363*(26), 2563.
6. Slutsky, A. S. (2010). Neuromuscular blocking agents in ARDS. *New England Journal of Medicine, 363*(12), 1176-1180.

**Different intervention or different population (5)**

1. Caldwell, J. E., Lau, M., & Fisher, D. M. (1995). Atracurium versus vecuronium in asthmatic patients: A blinded, randomized comparison of adverse events. *Anesthesiology, 83*(5), 986-991.
2. de Lemos, J. M., Carr, R. R., Shalansky, K. F., Bevan, D. R., & Ronco, J. J. (1999). Paralysis in the critically ill: Intermittent bolus pancuronium compared with continuous infusion. *Critical Care Medicine, 27*(12), 2648-2655.
3. Farenc, C., Lefrant, J. Y., Audran, M., & Bressolle, F. (2001). Pharmacokinetic-pharmacodynamic modeling of atracurium in intensive care patients. *Journal of Clinical Pharmacology, 41*(1), 44-50.
4. Farenc, C., Lefrant, J. -., Audran, M., Saissi, G., De La Coussaye, J. -., & Bressolle, F. (2000). Pharmacokinetics of atracurium and laudanosine in intensive care patients with acute respiratory distress syndrome undergoing mechanical ventilation. *Clinical Drug Investigation, 19*(2), 143-150. Forel, J. -., Roch, A., Marin, V., Michelet, P., Demory, D., Blache, J. -., et al. (2006). Neuromuscular blocking agents decrease inflammatory response in patients presenting with acute respiratory distress syndrome. *Critical Care Medicine, 34*(11), 2749-2757.
5. Hadbavny, A.; Rafkin, H.; Hoyt, J. Rocuronium infusions in the intensive care unit. European congress; 9th (1996; Sep : Glasgow). Intensive care medicine. 1996, 22 (3): 835-838
